# Hospital Dermatology: analysis of dermatological consultations in a tertiary teaching hospital^☆^

**DOI:** 10.1016/j.abd.2022.08.010

**Published:** 2023-05-08

**Authors:** Iago Gonçalves Ferreira, Camila Saraiva Almeida, Lucas Abascal Bulcão, Diego Gonçalves Ferreira, Magda Blessmann Weber, Renan Rangel Bonamigo

**Affiliations:** a Dermatology Service, Santa Casa de Misericordia de Porto Alegre, Porto Alegre, RS, Brazil; b Department of Dermatology, Federal University of Health Sciences of Porto Alegre, Porto Alegre, RS, Brazil; c Faculty of Medicine, University of Caxias do Sul, Caxias do Sul, RS, Brazil; d Faculty of Medicine, Federal University of Rio Grande do Sul, Porto Alegre, RS, Brazil

**Keywords:** Dermatology, Epidemiology, Health services research, Inpatients, Skin diseases, Skin and connective tissue diseases

## Abstract

**Background:**

In hospital settings, dermatology can offer substantial clinical support for the diagnosis and management of skin conditions, reducing morbidity and mortality. Thus, the study aimed to analyze the profile of referrals and consultations performed by the Dermatology Service of the Santa Casa de Misericordia de Porto Alegre, from August 2018 to January 2020.

**Methods:**

This study is descriptive, quantitative, and retrospective, conducted through data collection and review of medical records and referrals. The variables included were clinical data of referrals, in-patients profiles, dermatological diagnoses, complementary exams, therapeutic conduct, and recommended follow-ups.

**Results:**

A total of 1020 referrals were analyzed, which resulted in 641 consultations (328 men, 313 women). The most prevalent skin disease groups were ‘Dermatitis and Eczema’ (33.1%) and ‘Other infectious skin diseases (21.8%), while the most frequent ICD-10 were ‘Drug eruptions – L27’ (9.9%) and ‘Other and unspecified dermatitis – L30’ (6.6%). Corticoids were the most recommended treatments (27.7%), followed by antifungals (13.1%). ‘Consultation Discharge’ (44%) and ‘Outpatient’ Dermatology follow-up (27%) were the most frequent causes for ending consultation.

**Study limitations:**

Among the study limitations, the authors highlight its retrospective nature, with data analysis based on referrals and medical records, which may present inaccurate or incomplete information. In addition to this, the study may demonstrate a certain degree of subjectivity due to the review and interpretation process conducted by the researchers. However, the definition of objective criteria based on previous studies attenuates such possible bias. Furthermore, considering that the Dermatology teams are composed of a preceptor dermatologist and residents, the established diagnoses were not submitted to third-party verification, except in the cases of skin biopsies and cultures. Thus, the professional’s experience and skills may have influenced the dermatological diagnoses.

**Conclusions:**

These findings underlie the importance of Dermatology in hospital assistance, contributing to the management of a wide range of skin conditions.

## Introduction

Skin diseases affect millions of people globally and represent 15% to 30% of outpatient care in health systems.^1^, ^2^, ^3^ In the hospital environment, dermatoses also demonstrate a high prevalence, representing a significant cause of morbidity and potential risk of life, mainly due to the increased vulnerability of hospitalized patients.^1^, ^4^, ^5^ A study about hospitalizations in the United States demonstrated primary dermatological diseases as responsible for 0.47% of hospitals’ mortality rate, reaching up to 3.29% of mortality when associated with hospital complications.^6^

Hospital dermatoses have a wide range of clinical aspects, presenting as primary cutaneous disorders, secondary manifestations of systemic diseases, or being developed by hospital care, which may be termed tertiary cutaneous disorders.^4^, ^7^ Regardless of their clinical presentations, appropriate diagnosis and treatment represent the main components in handling these cases, considering their high prevalence, morbidity, and mortality.^7^, ^8^, ^9^

However, skin diseases are often identified and managed by non-dermatologists, especially internists and/or general practitioners, who in most cases have some degree of difficulty in interpreting skin lesions. Thus, they often establish inaccurate diagnoses, both for localized and generalized lesions, proposing inappropriate treatments.^8^, ^10^, ^11^ From this perspective, considering the prevalence and potential risk of skin conditions for inpatients, and their impact on the economics of health systems,^6^, ^7^, ^8^, ^9^ the authors emphasize the role of Dermatology for hospital assistance, considering that these specialists have the needed knowledge and skills to approach these clinical cases properly.^10^

In hospital settings, dermatologists’ assistance usually occurs as a consultation at the referral of other medical teams, aiming to clarify diagnoses and/or obtain therapeutic recommendations for skin disorders.^12^ Thus, in face of the role of Dermatology in hospital settings, as well as the burden of hospital dermatoses, it is essential to comprehend the nosological profile of hospital skin disorders and dermatological consultations profile. Therefore, this study aims to analyze the profile of dermatological consultations in the tertiary teaching hospital Santa Casa de Misericordia de Porto Alegre.

## Methods

### Scenario of the study

The hospital complex Santa Casa de Misericordia de Porto Alegre is formed by 7 hospitals in its main location: *Hospital Santa Clara* for adult assistance; *Hospital da Criança Santo Antônio* offers pediatric assistance, and other 5 hospitals specialized in cardiology (*Hospital São Francisco*), neurology and neurosurgery (*Hospital São José*), pneumology (*Pavilhão Pereira Filho*), oncology (*Hospital Santa Rita*) and transplants (*Hospital Dom Vicente Scherer*).^13^ The institution provides health assistance for the public health system (*Sistema Único de Saúde* ‒ SUS), which represents 73% of the total of patients, and for the supplementary health system (health plans and private), corresponding to the others 27%.^13^

The Dermatology Service consists of more than 40 dermatologists, two postgraduate programs (Residency Program of Dermatology and Specialization Course of Dermatology), and two Fellowships (Surgical Dermatology and Cosmetic Dermatology). The service develops an extensive range of assistance activities such as outpatient consultations, surgeries and cosmetic procedures, and inpatient consultation assistance in all sectors of the hospital complex.^13^

### Study design, data collection and processing

**Figure 1:**
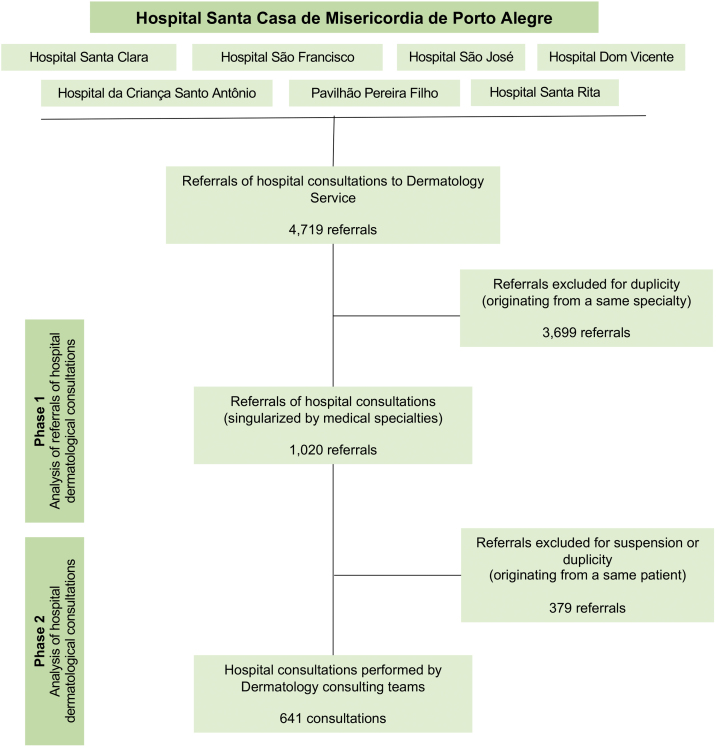
Flowchart of research methods * Referrals and consultations performed from August 2018 to January 2020.

This descriptive and observational study was performed under a retrospective analysis of hospital referrals and consultations of the Dermatology Service of Santa Casa de Porto Alegre from August 1, 2018, to January 31, 2020. The research was developed in two phases: analysis of referrals of hospital to dermatological consultations (phase 1) and analysis of hospital dermatological consultations (phase 2). From May to December 2021, the authors conducted the data collection through reviews of medical charts and database, utilizing the institution’s information system (Tasy®) as our data source. Fig. 1 represents the flowchart of research methods.

From the initial data collection, the authors identified 4,719 referrals to dermatological consultations, which were preliminarily analyzed, removing duplicate referrals from the same medical specialty (phase 1). These criteria were adopted due to the substantial number of referrals replicated by mistake or because of duplicate medical prescriptions. Thus, the consultation referrals and their respective medical charts records were analyzed, removing concurrent referrals from different specialties for the same patient, as well as consultations that did not occur due to suspension or loss (phase 2). Therefore, the authors aimed to identify the consultations which were effectively performed by the Dermatology team.

Based on a similar study reported by Huang and Chong,^8^ the authors arranged the set of variables in two groups: ‘profile of referrals’, hospital sector of origin, referring medical specialty, patient age and gender, clinical data, and diagnostic hypothesis reported by assistant teams; and ‘profile of dermatological consultations’, semiological description, recommended complementary exams, dermatological diagnoses, therapeutic recommendations, and proposed follow-ups.

Regarding the clinical data of referrals, the authors considered four essential clinical variables: morphology of skin lesions, distribution of skin lesions, time of clinical evolution, and diagnosis hypothesis or objective of referral. To systematize and standardize the medical record analysis, the authors classified the dermatologists’ diagnoses into categories and groups according to the International Classification of Diseases (ICD-10). In addition, to measure the repercussion of dermatological consultations, the authors included data from the total number of hospitalizations at the hospital complex and their respective dermatological diagnostic ICDs during the period covered by the study.

About the follow-up recommendations, the authors considered ‘Outpatient follow-up’ those dermatological consultations whose duration of Dermatology assistance was equal to or less than 7 days and with recommendation for outpatient follow-up after completion. On the other hand, ‘Inpatient + outpatient follow-up’ included those consultations whose Dermatology assistance duration was longer than 7 days and with a recommendation for outpatient follow-up after completion. The 7-day cutoff parameter was defined by the authors, based on findings from previous studies,^14^, ^15^ which revealed means of a length of stay hospitalizations for dermatological conditions ranging from 3 to 10 days, standing out a survey carried out by Orozco et al.,^16^ which revealed about 90% of dermatological hospitalizations lasted less than 7 days.

The categorical and numerical variables were analyzed descriptively, represented through dispersion measures, and absolute and relative frequencies. The comparative analysis between the groups of variables adopted the Chi-Square test (X²), considering a p-value ≤5% as statistically significant. The authors used the software IBM SPSS Statistics 23 to conduct the statistical data analysis.

## Results

**Figure 2:**
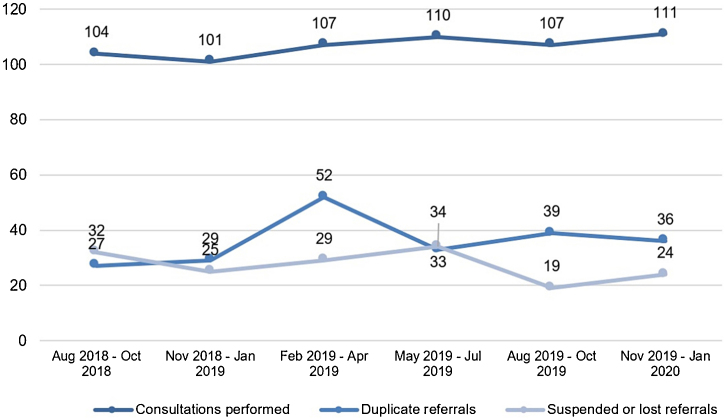
Flow of hospital dermatological consultations referrals from August 2018 to January 2020

Throughout the 18-month period, the authors identified 70,255 hospitalizations in the hospital complex Santa Casa de Porto Alegre. Among these, the authors verified 3,467 dermatological diagnostic ICDs, about 1 in every 20 hospitalizations. Regarding the consultation referrals, a total of 1,020 hospital consultations referrals were forwarded to the Dermatology Service. There was a monthly average of 56.6 referrals, highlighting the “February ‒ April/2019” quarter with 188 referrals (Fig. 2).

**Figure 3:**
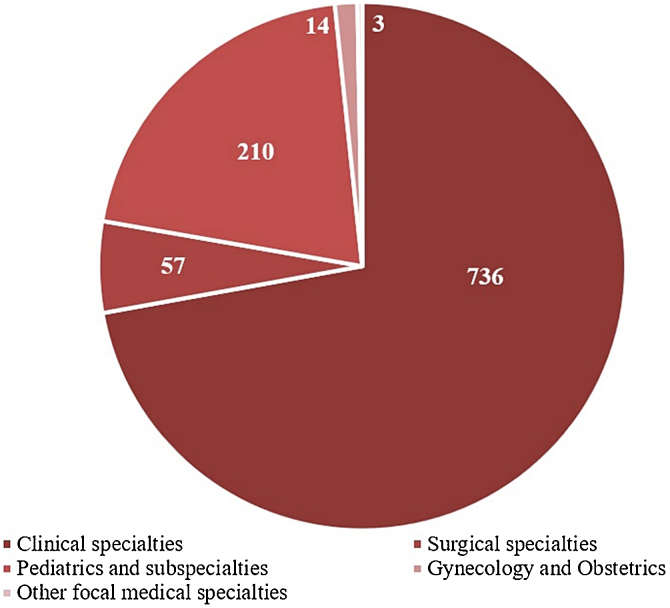
Referring medical areas of dermatological consultations

The ‘Clinical Ward’ sector and ‘Private insurance’ hospitalizations had the most frequent referrals, 51.7%, and 47.2%, respectively (Table 1). The medical specialties with the highest demands for dermatological consultations were ‘Internal medicine’ (25.2%), ‘Pediatrics’ (19%), and ‘Pneumology’ (8%), while ‘Clinical specialties’ and ‘Pediatrics and subspecialties’ were the main medical areas, with 72% and 21% of referrals, respectively (Fig. 3).

**Table 1:** Profile of hospital dermatological consultations referrals

Hospital consultations referrals		
Hospital	n	%
Clinical Hospitals^a^	566	55.5
Pediatric hospital^a^	266	26.1
Oncology Hospital^a^	98	9.6
Total	1020	100.0
**Hospital sector**	n	%
Clinical Ward	527	51.7
Surgical Ward	79	7.7
Pediatric Ward	257	25.2
Adult Emergency	63	6.2
Pediatric ICU	17	1.7
Adult ICU	69	6.8
Obstetric ward	8	0.8
Total	1020	100.0
**Sector character**	**n**	**%**
Public Health System	417	40.9
Private Health System	481	47.2
Mixed	122	12.0
Total	1020	100.0
**Distribution**^b^		
Monthly average	56.6 referrals	
Minimum (monthly)	43 referrals	
Maximum (monthly)	70 referrals	
Daily average	1.8 referrals	

**Values and percentages refer to variables, analyzed individually, in relation to a total of 1,020 hospital dermatological consultations referrals.

a Clinical Hospitals: Hospital Santa Clara (HSC), Hospital São Francisco (HSC), Pavilhão Pereira Filho (PPF), Hospital Dom Vicente Scherer (HDVS), Pediatric Hospital: Hospital da Criança Santo Antônio (HCSA), Oncology Hospital: Hospital Santa Rita (HSR).

b Values refer to hospital dermatological consultations referrals, singularized by specialty and by hospital admission.

In relation to clinical data, about half of the assistant teams’ referrals did not record any information about the patients’ dermatological conditions, and only 5.2% reported the four clinical data considered by research criteria. The location of skin lesions was the most referred clinical data (43.1%), while the time of clinical evolution was the least reported (10.8%). Only 28.5% (291/1020) of the referrals informed diagnostic hypotheses, which represent 199 records, excluding duplications. The diagnostic hypotheses reported by referring teams agreed with the diagnoses established by the Dermatology team in 61.8% (123/199) of the cases.

After the preliminary analysis and the exclusion of duplicate records, the authors observed 641 hospital dermatological consultations, with an average of 35.6 per month, most of which had a consultation response time of fewer than 24 hours. The Dermatology team established 727 dermatological diagnoses, which were classified into 15 groups of dermatoses according to the International Classification of Diseases (ICD-10). Most evaluations resulted in only 1 nosological diagnosis (85.3%), with a maximum of 3 diagnoses (1.3%) (Table 2).

**Table 2:** Profile of inpatient dermatological diagnoses and hospital dermatological consultations

Dermatological diagnoses and consultations				
**Recommended complementary exams**^a^				
		**n**		**%**
Skin biopsy		131		21.0
Bacterial culture		25		4.0
Direct mycological examination		43		6.9
Tzanck smear		7		1.1
Imaging exams		22		3.5
Laboratory exams		89		14.2
Total^a^		625^a^		100.0
**Dermatological diagnoses**				
*Frequency*				
		**n**		**%**
1 Diagnosis		535		85.3
2 Diagnoses		84		13.4
3 Diagnoses		8		1.3
Total^a^		625^a^		100.0
*Diagnostic concordance (Referring medical team × Dermatology team)*				
			**n**	**%**
Diagnostic hypothesis described (by referring medical team)		Yes	199	31.1
		No	442	68.9
Total		641		100.0
Concordance with dermatological diagnoses (by dermatology consulting team)		Yes	123	61.8
		No	76	38.2
Total		199	100.0	
**Skin diseases groups**				
		**n**	**%**	
Infectious dermatoses		206	28.3	
*Skin diseases groups*				
Infections of the skin and subcutaneous tissue		47	6.5	
Other infectious skin diseases		159	21.8	
*Groups by etiological agent*^b^				
Bacterial infections		57	7.8	
Viral infections		64	8.8	
Fungal infections		55	7.6	
Parasitic infections and infestations		30	4.1	
Bullous disorders		12	1.6	
Dermatitis and eczema		241	33.1	
Papulosquamous disorders		24	3.3	
Urticaria and erythema		16	2.2	
Radiation-related disorders of the skin and subcutaneous tissue		6	0.8	
Disorders of skin appendages		48	6.6	
Other disorders of the skin and subcutaneous tissue		73	10.0	
Other disorders of the skin and subcutaneous tissue (Non-L ICD)		28	3.8	
Neoplastic skin diseases (malignant)		29	3.9	
Neoplastic skin diseases (benign)		21	2.9	
Secondary malignant neoplasm of other sites (metastasis)		8	1.1	
General dermatological exam		9	1.2	
Congenital malformations with cutaneous involvement		6	0.8	
Total^c^		727^c^	100.0	
**Recommended treatments**^a^				
		n	%	
Recommended treatment	Yes	251	40.2	
	No	374	59.8	
*Antibiotics*				
Recommended	Yes	77	12.3	
	No	548	87.7	
Drug form	Topical	21	27.3	
	Systemic	56	72.7	
*Corticosteroids*				
Recommended	Yes	173	27.7	
	No	452	72.3	
Drug form	Topical	117	67.6	
	Systemic	56	32.4	
*Antifungals*				
Recommended	Yes	82	13.1	
	No	543	86.9	
Drug form	Topical	65	79.3	
	Systemic	17	20.7	
*Antiviral*				
Recommended	Yes	38	6.1	
	No	587	93.9	
Type of antiviral	Oral acyclovir	19	50.0	
	Intravenous acyclovir	18	47.4	
	Other antivirals	1	2.6	
*Antiparasitic*^d^				
Recommended	Yes	31	5.0	
	No	594	95.0	
Type of antiparasitic	Permethrin	3	9.7	
	Ivermectin	9	29.0	
	Permethrin + Ivermectin	12	38.7	
	Sulfur 6%	7	22.6	
*Moisturizer*				
Recommended	Yes	103	16.5	
	No	522	83.5	
Type of moisturizer	Specific	41	39.8	
	Generic	62	60.2	

a Total of dermatological consultations with diagnostic processes completed. excluding follow-up losses in the beginning of Dermatology assistance.

b Skin infections and ICDs: bacterial infections (L00. L01. L02. L03. L08. A30. A46. A53); viral infections (B00. B01. B02. B07. B08. B97); fungal infections (B35. B36. B37. B42); Parasitic infections and infestations (B85. B86. B87).

c Total of diagnoses established by hospital dermatological consultations.

d Antiparasitic drugs for treatment of scabies.

**Figure 4:**
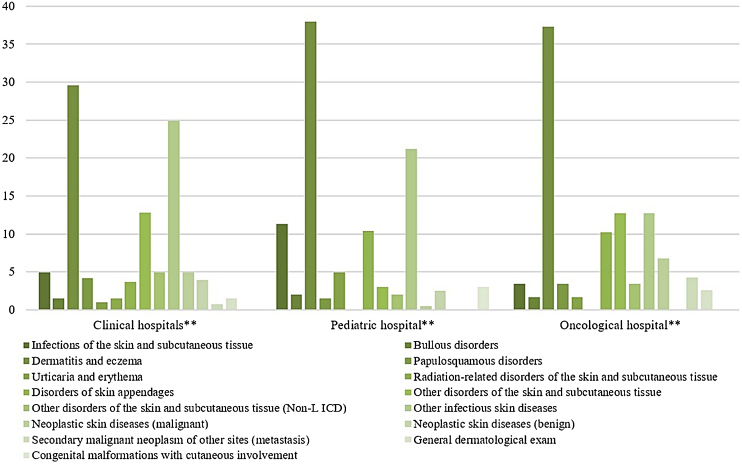
Skin diseases groups of dermatological consultations. by hospitals. from August 2018 to January 2020*. * Chi-Square Test (X² = 114.754). p < 0.001 ** Clinical Hospitals: Hospital Santa Clara (HSC). Hospital São Francisco (HSC). Pavilhão Pereira Filho (PPF). Hospital Dom Vicente Scherer (HDVS). Pediatric Hospital: Hospital da Criança Santo Antônio (HCSA). Oncology Hospital: Hospital Santa Rita (HSR).

**Figure 5:**
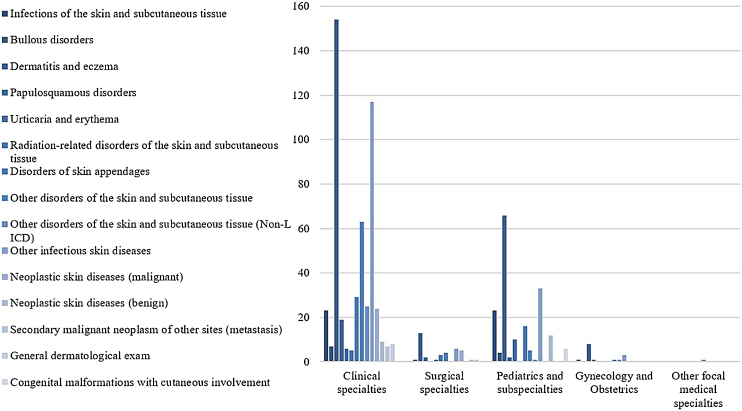
Skin diseases groups of dermatological consultations. by referring medical specialties. from August 2018 to January 2020* * Chi-Square Test (X² = 147.882). p < 0.00).

The most prevalent groups of skin diseases were ‘Dermatitis and eczema’ (33.1%) and ‘Other infectious skin diseases (21.8%) (Table 2). They also were the prevailing groups in the ‘Clinical Hospitals’ and the ‘Pediatric Hospital’ (Fig. 4), also in Clinical and Pediatric specialties (Fig. 5). ‘Dermatitis due to substances taken internally – L27’ emerges as the main dermatosis (9.9%), mainly in ‘Clinical Hospitals’ and in age groups ‘26–35 years’, ‘36–45 years’ and ‘56–65 years’ (Table 3, Table 4). Among the pediatric population, ‘Atopic Dermatitis – L20’ stands out, with 20.8% of dermatological conditions in the age group ‘2–12 years’ and 15.8% in the ‘Pediatric Hospital’, as well as ‘Scabies – B86’, which represented 16.7% of diagnoses among children under 1-year-old (Table 4).

**Table 3:** Summary of major diagnostic ICDs of dermatological consultations

Major diagnostic ICDs^a^			
**Top 15 dermatological diagnoses**		**n**	**%**
L27 – Dermatitis due to substances taken internally		72	9.9
L30 – Unspecified dermatitis		48	6.6
B00 – Herpesvirus infections		38	5.2
L20 – Atopic dermatitis		34	4.7
B86 – Scabies		28	3.8
B35 – Dermatophytosis		26	3.6
L21 – Seborrheic dermatitis		26	3.6
L01 – Impetigo		25	3.4
L40 – Psoriasis		23	3.2
L25 – Contact dermatitis		21	2.9
C44 – Other malignant neoplasm of skin		21	2.9
B37 – Candidiasis		19	2.6
L29 – Pruritus		15	2.1
L70 – Acne		14	1.9
L85 – Other epidermal thickening		12	1.6
**Hospitals**		**n**	**%**
**Clinical hospitals**^b^	L27 – Dermatitis due to substances taken internally	37	9.1
	L30 – Unspecified dermatitis	33	8.1
	B00 – Herpesvirus infections	25	6.1
	B35 – Dermatophytosis	20	4.9
	C44 – Other malignant neoplasm of skin	16	3.9
**Pediatric hospital**^b^	L20 – Atopic dermatitis	32	15.8
	L27 – Dermatitis due to substances taken internally	23	11.3
	L01 – Impetigo	18	8.9
	B86 – Scabies	17	8.4
	L70 – Acne	10	4.9
**Oncology hospital**^b^	L27 – Dermatitis due to substances taken internally	12	10.2
	L30 – Unspecified dermatitis	9	7.6
	L21 – Seborrheic dermatitis	7	5.9
	L25 – Contact dermatitis	5	4.2
	C44 – Other malignant neoplasm of skin	5	4.2

a International Classification of Diseases (ICD-10).

b Clinical Hospitals: Hospital Santa Clara (HSC). Hospital São Francisco (HSC). Pavilhão Pereira Filho (PPF). Hospital Dom Vicente Scherer (HDVS). Pediatric Hospital: Hospital da Criança Santo Antônio (HCSA). Oncology Hospital: Hospital Santa Rita (HSR).

**Table 4:** Major diagnostic ICDs of dermatological consultations. by age groups

Major diagnostic ICDs			
Age groups		n	%
0–1 year	B86 – Scabies	11	16.7
	L21 – Seborrheic dermatitis	9	13.6
	L20 – Atopic dermatitis	7	10.6
	D18 – Hemangioma and lymphangioma	6	9.1
	L01 – Impetigo	6	9.1
2–12 years	L20 – Atopic dermatitis	20	20.8
	L27 – Dermatitis due to substances taken internally	12	12.5
	L01 – Impetigo	10	10.4
	B86 – Scabies	6	6.2
	L21 – Seborrheic dermatitis	5	5.2
13–18 years	L27 – Dermatitis due to substances taken internally	12	21.8
	L70 – Acne	7	12.7
	B00 – Herpesvirus infections	6	10.9
	L20 – Atopic dermatitis	5	9.0
	L01 – Impetigo	3	5.4
19–25 years	L02 – Cutaneous abscess. furuncle and carbuncle	4	19.0
	B08 – Molluscum contagiosum	2	9.5
	B35 – Dermatophytosis	2	9.5
	B00 – Herpesvirus infections	1	4.8
	B01 – Varicella	1	4.8
26–35 years	L27 – Dermatitis due to substances taken internally	7	15.2
	L21 – Seborrheic dermatitis	5	10.9
	L30 – Unspecified dermatitis	3	6.5
	B00 – Herpesvirus infections	2	4.3
	B97 – Papillomavirus infections	2	4.3
36–45 years	L27 – Dermatitis due to substances taken internally	8	13.6
	B00 – Herpesvirus infections	5	8.5
	L30 – Unspecified dermatitis	5	8.5
	B35 – Dermatophytosis	4	6.8
	L25 – Contact dermatitis	4	6.8
46–55 years	B00 – Herpesvirus infections	6	9.3
	L27 – Dermatitis due to substances taken internally	6	9.3
	B35 – Dermatophytosis	5	7.7
	L25 – Contact dermatitis	4	6.1
	L40 – Psoriasis	4	6.1
56–65 years	L27 – Dermatitis due to substances taken internally	11	8.5
	C44 – Other malignant neoplasm of skin	9	7.0
	L30 – Unspecified dermatitis	9	7.0
	L40 – Psoriasis	7	5.4
	B35 – Dermatophytosis	5	3.9
66–85 years	L30 – Unspecified dermatitis	19	11.0
	L27 – Dermatitis due to substances taken internally	12	6.9
	B00 – Herpesvirus infections	11	6.4
	B37 – Candidiasis	9	5.2
	C44 – Other malignant neoplasm of skin	8	4.6
>85 years	C44 – Other malignant neoplasm of skin	3	17.6
	L27 – Dermatitis due to substances taken internally	3	17.6
	L30 – Unspecified dermatitis	2	11.8
	B36 – Superficial mycoses	1	5.9
	B37 – Candidiasis	1	5.9

On infectious dermatoses, the viral etiology (8.8%) stands out, followed by bacterial (7.8%), fungal (7.6%), and parasitic (4.1%) infections (Table 2). ‘Bacterial cultures’ were requested in only 4% of the consultations and had *Staphylococcus aureus* as the main etiological agent (16%; 4/25). The ‘direct mycological examination’ was performed in 6.9% of the evaluations, with negative results in 60.4% of the samples (26/43) and dermatophytes as the most prevalent fungal agent (20.9%; 9/43).

The most recommended complementary exams were skin biopsies (21%), which presented anatomopathological reports in concordance with the clinical diagnoses of Dermatology teams in 58.8% of the evaluations (77/131) and changed the final diagnosis in 33.3% of cases (40/131). The anatomopathological reports were inconclusive in 9.9% of the samples (13/131), and the loss of materials corresponded to 8.4% (11/131).

Regarding the therapeutic management, corticoids were the most recommended drugs in consultations (27.7%), with a predominance of topical presentations (67.6%). Antibiotics were indicated in 12.3% of the evaluations, highlighting the systemic presentation (56%), mainly cephalexin (26%, 20/77) and oxacillin (16.9%, 13/77). Antifungals were recommended in 13.1% of the Dermatology evaluations. Among them, the authors highlight the topical presentations (79.3%), the azole derivatives ketoconazole (45.1%; 37/82), and miconazole (8.5%; 7/82). Antivirals and antiparasitics were less frequent among the recommended prescriptions, with 6.1% and 5.0%, respectively (Table 2).

A significant majority of the dermatological consultations (70.8%; 454/641) required hospital follow-up for less than 7 days, consisting of ‘Discharges of consultation’ (44.3%; 284/641) and ‘Outpatient follow-up’ (27,5%; 170/641). On the other hand, 22.8% of consultations (146/641) required hospital follow-up longer than 7 days (‘Inpatient follow-up + Outpatient follow-up’). Furthermore, 6.4% (41/641) of dermatological consultations presented loss of follow-up, 16 (2.5%) during the diagnostic process, and 25 (3.9%) after the diagnostic definition by the Dermatology team.

## Discussion

In the hospital context, skin diseases are highly prevalent, whether or not they are the primary cause of hospitalization.^1^, ^4^, ^5^, ^17^^,^^18^ Thus, our findings evidenced that dermatoses have a prevalence of 4.9% among inpatients, a greater proportion than the results reported in an investigation about hospitalizations in the Brazilian public health system in 2019, which presents diseases of the skin and subcutaneous tissue representing approximately 2.5% of the total hospitalizations.^19^ This difference may be related to the parameter adopted in the present study, which considered all ICDs recorded during hospital care, not just those attributed as causes of hospitalization.

### Referrals of hospital dermatological consultations

Usually, dermatologists work in hospital care in the form of consultations requested by the assistant teams, thus contributing to the diagnosis and clinical management of skin disorders.^4^, ^20^, ^21^ Consultation referrals must present basic information about the clinical conditions of hospitalized patients,^12^ considering that the characteristics of the lesions and the clinical evolution are essential elements for the dermatological diagnosis.^10^

However, despite the relevance of an adequate semiological description, there was a significant lack of data in the analyzed referrals, especially regarding the morphology of skin lesions, converging with previous studies.^1^, ^20^, ^22^, ^23^, ^24^ According to Fayne et al.^24^ and Lorente-Lavirgen et al.,^20^ non-dermatologist physicians usually describe skin lesions with non-specific or generic terms, such as ‘rash’, affecting the quality of referrals. Therefore, the authors reinforce the need to improve medical training in Dermatology, both in medical undergraduate and residency programs of other specialties.^25^

“Internal Medicine” presented the highest number of referrals to Dermatology, which corroborates the findings of other authors.^5^, ^8^, ^10^, ^12^^,^^26^, ^27^, ^28^ The greater demand observed in this specialty may be related to its significant generalist and backup nature, as well as its high volume of assisted patients. As for the other medical specialties, the literature shows divergences in their demands, standing out in second place ‘Pediatrics’,^12^, ^26^ as observed in this study; ‘Surgery’,^1^, ^10^, ^29^ ‘Hematology’,^20^ ‘Neurology’,^5^ and ‘Emergency Medicine’^24^ (Table 5).^1^, ^5^, ^8^, ^10^^,^^12^, ^14^, ^15^, ^20^^,^^22^, ^23^, ^24^, ^26^, ^27^, ^28^, ^29^, ^30^, ^31^ In addition, as reported by Galimberti et al.,^10^ clinical specialties presented more referrals than surgical ones. This can relate to the inpatient profile or even indicate a probable inattention to skin disorders from surgeons. This situation will require further studies for a better understanding.

**Table 5:** Literature review about Hospital Dermatology assistance

Author (year)	Institution (country)	Duration	Casuistry	Major referring medical specialties (%)	Major skin diseases groups (%)
Ferreira et al.	Santa Casa de Misericordia de Porto Alegre (Brazil)	18 months	641	Internal medicine (22.5)	Dermatitis and eczema (33.1)
				Pediatrics (22.1)	Skin infections (28.3)
				Nephrology (7.9)	Other disorders of the skin and subcutaneous tissue (10.0)
Huang e Chong (2015)^8^	Khoo Teck Puat Hospital (Singapore)	4 months	168	Internal medicine (72.6)	Skin infections (32)
				Geriatrics (12.5)	Inflammatory dermatoses (32)
				Orthopedics (6.0)	Drug eruptions (10.5)
Samorano-Lima et al. (2014)^30^	Hospital das Clínicas da Faculdade de Medicina da Universidade de São Paulo (Brazil)	8 years	3.308	Uninformed^a^	Contact dermatitis (17.5)
					Skin infections (15.9)
					Bullous dermatoses (10.9)
Cavero-Guardamino (2017)^12^	Hospital Nacional Guillermo Almenara Irigoyen (Peru)	5 years	4.479	Internal medicine (21.2)	Inflammatory dermatoses (29.1)
				Pediatrics (9.2)	Skin infections (26.6)
				Psychiatry (8.7)	Papulosquamous dermatoses (8.8)
Storan et al. (2014)^27^	Mayo Clinic (USA)	12 years	614	Internal medicine (30.3)	Skin infections (18.5)
				Hematology and oncology (20.3)	Dermatitis (17.8)
				Surgery (13.6)	Drug eruptions (12.9)
Alani et al. (2016)^1^	University Hospital Limerick (Ireland)	9 months	220	Internal medicine (45.0)	Eczema (21.8)
				Surgery (25.0)	Psoriasis (7.3)
				Pediatrics (23.6)	Cellulitis (5.9)
Can et al. (2014)^26^	Goztepe Research and Training Hospital (Turkey)	18 months	282	Internal medicine (34.7)	Drug eruptions (10.9)
				Pediatrics (29.8)	Superficial cutaneous mycoses (8.1)
				Neurology (8.9)	Contact dermatitis (7.8)
Chavez-Alvarez et al. (2019)^28^	Hospital Universitario “Dr. José Eleuterio González” ‒ Universidad Autônoma de Nuevo León (Mexico)	3 years	1.059	Internal medicine (75.0)	Drug eruptions (13.0)
				Neurosurgery (6.4)	Adnexal diseases (11.9)
				Surgery (5.8)	Viral infections (10.0)
Özyurt et al. (2014)^22^	Izmir Atatürk Training and Research Hospital (Turkey)	5 months	417	Internal medicine (17.3)	Contact dermatitis (9.4)
				Neurology (9.8)	Fungal infections (8.4)
				Gynecology and obstetrics (8.4)	Drug eruptions (6.7)
Fernandes et al. (2012)^29^	Hospital de Santo António. Porto Hospital Center (Portugal)	12 months	217	Internal medicine (33.7)	Skin infections (33.2)
				Surgery (10.3)	Eczema (9.5)
				Pediatrics (8.9)	Drug eruptions (7.3)
Lorente-Lavirgen et al. (2012)^20^	Hospital Virgen del Rocío (Spain)	12 months	429	Internal medicine (26.8)	Inflammatory dermatoses (35.79)
				Hematology (15.4)	Skin infections (25.72)
				Nephrology (10.2)	Bullous and autoimmune dermatoses (10.5)
Galimberti et al. (2016)^10^	Cleveland Clinic Lerner College of Medicine (USA)	12 months	691	Internal medicine (45.0)	Drug eruptions (13.0)
				Surgery (12.0)	Contact dermatitis (8.5)
				Hematology and oncology (9.0)	Viral infections (5.8)
Tay et al. (2010)^31^	Singapore General Hospital (Singapore)	12 months	731	Internal medicine (36.0)	Eczema and Dermatitis (33.1)
				Nephrology (10.4)	Skin infections (23.4)
				Surgery (7.8)	Drug eruptions (12.3)
Mancusi e Neto (2010)^5^	Hospital das Clínicas da Faculdade de Medicina da Universidade de São Paulo (Brazil)	4 months	313	Internal medicine (24)	Skin infections (26.8)
				Neurology (12)	Eczematous dermatoses (16.6)
				Cardiology (11)	Drug eruptions (14.0)
Bale e Chee (2014)^14^	John Hunter Hospital (Australia)	12 months	97	Pediatrics (12.0)	Inflammatory dermatoses (59.8)
				Immunology (9.0)	Vasculopathies (14.4)
				Internal medicine/ Nephrology Infectology/ Ophthalmology (8.0)	Other dermatoses (9.3)
Connolly. e Silverstein (2015)^23^	Stony Brook University Medical Center (USA)	13 months	243	Internal medicine (45.7)	Skin infections (24.0)
				Intensive medicine (11.5)	Drug eruptions (22.3)
				Hematology and oncology (9.9)	Inflammatory dermatoses (21.0)
Fayne et al. (2020)^24^	University of Miami Miller School of Medicine (USA)	6 years	812	Internal medicine (55.6)	Bacterial infections (11.4)
				Emergency medicine (21.9)	Viral infections (6.0)
				Surgery (6.5)	Drug eruptions (5.4)
Zhao et al. (2016)^15^	St George Hospital (Australia)	12 months	219	Clinical specialties (68.0)	Dermatitis (30.9)
				Surgical Specialties (16.3)	Skin infections (27.5)
				Intensive medicine (7.3)	Skin neoplasms (6.4)

*Data not informed by authors.

a The literature review was performed in data bases: Medline/Pubmed. Lilacs and Scielo. adopting the search strategy: ‘Dermatology’ AND ‘Hospital’ OR ‘Inpatients’. with a ‘last 10-years’ filter (2010‒2020). Based on the preliminary data collection. the authors proceeded an exploratory reading of publications’ titles and abstracts. using as inclusion criteria: studies with a casuistry of hospitalized populations. presenting skin conditions as a primary or secondary cause of hospitalization. assisted by Dermatology teams. and with population groups not segmented or limited by medical field. hospital sector and/or comorbidity.

Another relevant aspect consisted of the expressive amount of suspended or duplicated consultation referrals ‒ around 40% ‒ which may be related to communication difficulties among medical specialties teams, as well as the inadequacy of consultation referral flows to Dermatology. According to Alani et al.,^1^ dermatological consulting processes have their weaknesses, often presenting incomplete information and/or misdiagnoses, reinforcing the need of referral flows reorganization. In view of this, Prada-Garcia et al.^32^ observed that 74% of consulting referrals were requested again by the assistant teams within less than 48 hours of the first request, thus generating duplications.

Regarding the suspensions, these may be related to the lack of clinical criteria for hospital dermatological consultations, causing their suspension or even the patients’ discharge before the dermatologists’ evaluation. From this perspective, Mancusi et al.^5^ highlighted that 21% of dermatological referrals did not have severity criteria compatible with dermatological evaluation in hospital settings.

### Dermatological consultations in hospital settings

The dermatology team conducted 641 consultations over the 18-month period of study, with an annual average of 427 evaluations. This amount is in line with findings from other tertiary hospitals in Brazil,^30^ Spain,^20^ and Singapore.^8^ However, the result differs from hospital services in other countries, such as the United States^10^, ^21^, ^27^ and Peru,^12^ which may indicate a higher influence of intrinsic characteristics of hospitals than the macroeconomic and social conjuncture of these countries.

A considerable amount of the dermatological evaluations resulted in a single diagnosis (85.3%). Although in different proportions, this trend was also verified by Huang and Chong^8^ (52.4%), Fayne et al.^24^ (62.0%), Alani et al.^1^ (63.0%), and Samorano-Lima et al.^30^ (85.4%), evidencing the need for better understanding of that fact. ‘Dermatitis and eczema’ were the most frequent skin disease group, followed by infectious dermatoses ‒ represented by the groups ‘Infections of the skin and subcutaneous tissue’ and ‘Other infectious skin diseases’.

Despite the diversity of methods found in the literature, the authors noted comparable nosological profiles of hospital dermatoses in similar studies (Table 5),^1^, ^5^, ^8^, ^10^^,^^12^, ^14^, ^15^, ^20^^,^^22^, ^23^, ^24^, ^26^, ^27^, ^28^, ^29^, ^30^, ^31^ indicating a predominance of acute and inflammatory skin conditions. Considering that such clinical conditions are important factors of morbidity and mortality,^6^ reducing survival and increasing the length of hospital stay, Dermatology can substantially contribute to inpatient care.

Considering a similar investigation,^33^ conducted within the scope of Primary and Secondary Health Care in a capital city in southern Brazil, the authors identified a confluence among the profile of skin diseases in Primary Care and the tertiary hospitals of the present study. From this perspective, ‘Dermatitis and eczema’ and ‘Other infectious skin diseases’ stand out as the main dermatoses groups in both scenarios. However, they denote different frequencies: ‘Dermatitis and eczema’ 33.1% vs. 21.5%^33^ and ‘Other infectious skin diseases’ 21.8% vs. 25.6%.^33^

In face of the convergence among Primary and Tertiary Care profiles, a reasonable explanation may be the generalist nature of both scenarios, although they present particularities related to medical care practices and assisted population. Whereas in Primary Care^33^ ‘Atopic dermatitis’ (6.4%), ‘Other disorders of the skin and subcutaneous tissue’ (5.1%), and ‘Scabies’ (4.5%) appeared as the main dermatological diseases, our findings showed ‘Dermatitis due to substances taken internally’ (9.9%), ‘Unspecified dermatitis’ (6.6%), and ‘Herpesvirus infections’ (5.2%) as the most prevalent disorders among inpatients, conditions intrinsically associated with hospital care and states of immunosuppression.

Considering particular ICD dermatological diagnoses, the authors highlighted the preponderance of ‘Dermatitis related to substances taken internally’ (ICD L27) ‒ also denominated ‘drug eruptions’ or ‘cutaneous adverse drug reactions’, presenting high prevalence in most age groups, with peaks in ages ‘26‒45 years’ and ‘56–65 years’. Such findings are in accordance with the profile of hospital dermatoses in other institutions: Goztepe Research and Training Hospital in Turkey^26^ (10.9%); Jackson Memorial Hospital^24^ (9.9%), Mayo Clinic^27^ (12.9%) and Cleveland Clinic^10^ (13.0%) in the United States; University Hospital “*Dr. Jose Eleuterio Gonzalez*”^28^ (13%) in México; and Singapore General Hospital^31^ (12.3%) in Singapore.

During hospitalization, recurrent exposition to systemic medications ‒ especially analgesics, non-steroidal anti-inflammatories, and neuroleptics ‒ as well as debilitated patients’ conditions consist of significant risk factors for drug skin disorders.^5^, ^34^ Thus, the risks of irrational prescriptions inwards need to be alerted to assistant medical teams.

Another relevant aspect refers to the predominance of infectious dermatoses, such as scabies and impetigo, and inflammatory skin disorders, such as seborrheic dermatitis and atopic dermatitis, among children and adolescents. These findings agreed with previous studies,^16^, ^32^, ^35^ which pointed out infectious and inflammatory dermatoses as the main skin conditions among inpatients under 18 years old. Skin fragility and immunological immaturity of patients in this age group may be likely explanations for these results.

As for the complementary investigation, converging with similar studies,^10^, ^23^, ^24^, ^26^^,^^29^ skin biopsies were the most recommended exams, being performed in 21% of the consultations. This proportion, however, proved to be lower than the findings of other authors: Galimberti et al.^10^ (30.8%), Lorente-Lavirgen et al.^20^ (35.4%), Fernandes et al.^29^ (34.8%), Huang et al.^8^ (31.0%) and Fayne et al.^24^ (41.2%). This difference may be related to varying degrees of severity and/or complexity of skin disorders among the studies, or it may correspond to the clinical experience and clinical management practices of the services. Thus, the authors emphasize the concordance rate of 58.8% between clinical diagnoses and pathological reports, which differs from the rates of the Cleveland Clinic^10^ (71.9%).

The consulting team recommended mostly corticoids (27.1%), mainly in the topical form, reinforcing the inflammatory and acute character of the skin disorders. In most consultations, the Dermatology team did not recommend immediate therapeutic approaches (60.8%), in contrast to similar studies,^10^, ^24^ which suggests that such cases were residual or resolving conditions, or could be followed up on an outpatient basis, not requiring interventions during hospitalization.

Regarding the follow-up recommendations, outpatient follow-up was recommended in 49.3% (316/641) of dermatological consultations, similar to the findings at the Jackson Memorial Hospital (55.0%),^24^ but higher than reported in the University Limerick Hospital^1^ (33.0%).

## Conclusion

From the study findings, the authors outlined the profile of referrals and consultations in Dermatology, highlighting the impact of this medical specialty on inpatient care. With regard to referrals, there was a significant lack of clinical data, particularly the semiological description of skin lesions, and a predominance of ‘Internal Medicine’ and ‘Pediatrics’ as the most referring medical specialties, which have essentially a generalist nature. A considerable portion of the referrals demonstrated clinical criteria inconsistent with tertiary hospital care. The establishment of flows and referral protocols for dermatological consultations could solve this issue.

In dermatological consultations, the authors observed the relevance of clinical dermatological examination for skin disease diagnosis, insofar as verified a reduced proportion of complementary exams requested. The most prevalent groups of skin diseases were ‘Dermatitis and eczema’, especially drug eruptions, as well as infectious dermatoses, acute conditions that, in most cases, require agile and accurate clinical management, reinforcing the importance of dermatologists in hospital care.

## Ethical approval

Approval was obtained in the Comitê de Ética em Pesquisa da Santa Casa de Misericórdia de Porto Alegre (Registration number: 34192820.9.0000.5335 / Report: 5.076.702).

## Financial support

None declared.

## Authors’ contributions

Iago Gonçalves Ferreira: Conception and planning of the study; critical review of the literature; data collection, analysis and interpretation; effective participation in research orientation; critical review of the manuscript; manuscript preparation and writing; statistical analysis; approval of the final version of the manuscript.

Camila Saraiva Almeida: Conception and planning of the study; critical review of the literature; data collection, analysis and interpretation; critical review of the manuscript; manuscript preparation and writing; statistical analysis; approval of the final version of the manuscript.

Lucas Abascal Bulcão: Conception and planning of the study; critical review of the literature; data collection, analysis and interpretation; critical review of the manuscript; manuscript preparation and writing; statistical analysis; approval of the final version of the manuscript.

Diego Gonçalves Ferreira: Data collection, analysis and interpretation; manuscript preparation and writing; statistical analysis; approval of the final version of the manuscript.

Magda Blessmann Weber: Conception and planning of the study; critical review of the literature; effective participation in research orientation; critical review of the manuscript; approval of the final version of the manuscript.

Renan Rangel Bonamigo: Conception and planning of the study; critical review of the literature; effective participation in research orientation; critical review of the manuscript; approval of the final version of the manuscript.

## Conflicts of interest

None declared.
